# Impact of ivermectin components on *Anopheles dirus* and *Anopheles minimus* mosquito survival

**DOI:** 10.1186/s13071-024-06294-6

**Published:** 2024-05-15

**Authors:** Pattarapon Khemrattrakool, Thitipong Hongsuwong, Phornpimon Tipthara, Rattawan Kullasakboonsri, Theerawit Phanphoowong, Patchara Sriwichai, Borimas Hanboonkunupakarn, Podjanee Jittamala, Joel Tarning, Kevin C. Kobylinski

**Affiliations:** 1 Mahidol Oxford Tropical Medicine Research Unit, Faculty of Tropical Medicine, Mahidol University, Bangkok, Thailand; 2 Department of Medical Entomology, Faculty of Tropical Medicine, Mahidol University, Bangkok, Thailand; 3 Department of Clinical Tropical Medicine, Faculty of Tropical Medicine, Mahidol University, Bangkok, Thailand; 4 Department of Tropical Hygiene, Faculty of Tropical Medicine, Mahidol University, Bangkok, Thailand; 5 Centre for Tropical Medicine and Global Health, Nuffield Department of Clinical Medicine, University of Oxford, Oxford, UK

**Keywords:** *Anopheles*, Racemic ivermectin, Ivermectin B_1a_, Ivermectin B_1b_, Survival

## Abstract

**Background:**

Ivermectin mass drug administration to humans or livestock is a potential vector control tool for malaria elimination. Racemic ivermectin is composed of two components, namely a major component (> 80%; ivermectin B_1a_), which has an ethyl group at C-26, and a minor component (< 20%; ivermectin B_1b_), which has a methyl group at C-26. There is no difference between the efficacy of ivermectin B_1a_ and ivermectin B_1b_ efficacy in nematodes, but only ivermectin B_1b_ has been reported to be lethal to snails. The ratios of ivermectin B_1a_ and B_1b_ ratios in ivermectin formulations and tablets can vary between manufacturers and batches. The mosquito-lethal effects of ivermectin B_1a_ and ivermectin B_1b_ have never been assessed. As novel ivermectin formulations are being developed for malaria control, it is important that the mosquito-lethal effects of individual ivermectin B_1a_ and ivermectin B_1b_ compounds be evaluated.

**Methods:**

Racemic ivermectin, ivermectin B_1a_ or ivermectin B_1b_, respectively, was mixed with human blood at various concentrations, blood-fed to *Anopheles dirus* sensu stricto and *Anopheles minimus* sensu stricto mosquitoes, and mortality was observed for 10 days. The ivermectin B_1a_ and B_1b_ ratios from commercially available racemic ivermectin and marketed tablets were assessed by liquid chromatography-mass spectrometry.

**Results:**

The results revealed that neither the lethal concentrations that kills 50% (LC_50_) nor 90% (LC_90_) of mosquitoes differed between racemic ivermectin, ivermectin B_1a_ or ivermectin B_1b_ for *An. dirus* or *An. minimus*, confirming that the individual ivermectin components have equal mosquito-lethal effects. The relative ratios of ivermectin B_1a_ and B_1b_ derived from sourced racemic ivermectin powder were 98.84% and 1.16%, respectively, and the relative ratios for ivermectin B_1a_ and B_1b_ derived from human oral ivermectin tablets were 98.55% and 1.45%, respectively.

**Conclusions:**

The ratio of ivermectin B_1a_ and B_1b_ does not influence the *Anopheles* mosquito-lethal outcome, an ideal study result as the separation of ivermectin B_1a_ and B_1b_ components at scale is cost prohibitive. Thus, variations in the ratio of ivermectin B_1a_ and B_1b_ between batches and manufacturers, as well as potentially novel formulations for malaria control, should not influence ivermectin mosquito-lethal efficacy.

**Graphical abstract:**

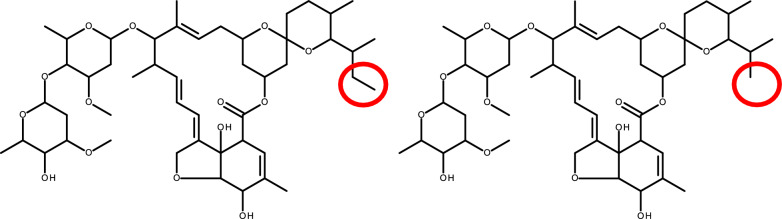

## Background

Ivermectin mass drug administration (MDA) is a potential new tool for malaria control and elimination. Blood-feeding on ivermectin-treated humans or livestock is lethal to all *Anopheles* mosquitoes investigated to date [1]. It was recently demonstrated that metabolites of ivermectin also have mosquito-lethal effects, with several metabolites possessing effects equal to the parent ivermectin compound [2, 3]. The glutamate-gated chloride (GluCl) ion channel is purported to be the primary target of ivermectin, with channel opening leading to chloride continuously flowing through the channel, ultimately causing flaccid paralysis of the musculature and possible death of the nematode or arthropod [4–6]. The results from an earlier protein crystallography study with the nematode *Caenorhabditis elegans* GluCl channel indicated that the first ivermectin sugar ring at C-13 is critical for initial binding to the GluCl M2-M3 loop, with subsequent opening and activation of the GluCl channel [7].

**Fig. 1:**
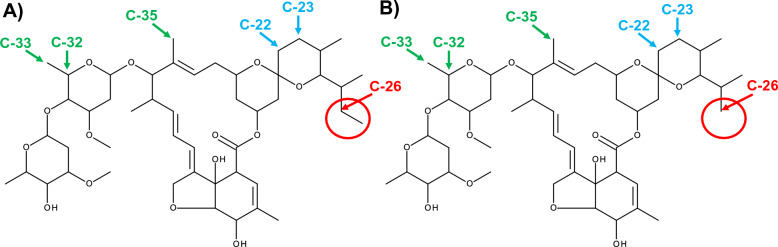
Molecular structures of ivermectin B_1a_ and ivermectin B_1b_. **A**,** B** Ivermectin B_1a_ (**A**) has an ethyl group at C-26 while ivermectin B_1b_ (**B**) has a methyl group at C-26, shown in red circles. The C-22 and C-23 single-bond hydrogenation point is shown in blue. The binding points of ivermectin to the M2–M3 loop of the glutamate-gated chloride ion channel are shown in green

Ivermectin belongs to a group of closely related macrocyclic lactones known collectively as the avermectins. Avermectin is the natural product of *Streptomyces avermitilis*. Avermectins comprise a series of pentacyclic macrolactones attached to a disaccharide of the methylated deoxysugar l-oleandrose at C-13. Fermentation of *S. avermitilis* produces four pairs of homologous compounds named: A_1a_, A_1b_; A_2a_, A_2b_; B_1a_, B_1b_; and B_2a_, B_2b_ [8]. The A series has a 5″-methoxyl group, while the B series has a 5″-hydroxyl group, which makes the B series more active [9–11]. Avermectin B_1_ differs from avermectin B_2_ in that avermectin B_1_ has an olefin double bond between C-22 and C-23 while avermectin B_2_ has a single bond between C-22 and C-23 and a hydroxy group rather than a hydrogen at C-23. This hydoxyl group leads to superior efficacy [12] and safety characteristics of avermectin B_1_, illustrating that minor changes in structure can lead to different efficacy outcomes [13]. The avermectin A_1_, A_2_, B_1_, B_2_ components are readily separated by chromatography, allowing for the separation of the superior B_1_ component at scale. The natural product of avermectin B_1_ is marketed as abamectin. Synthetic hydrogenation of avermectin B_1_ creates 22,23-dihydroavermectin with a single bond between C-22 and C-23 (Fig. 1) and is marketed as ivermectin. Ivermectin B_1_ has major (ivermectin B_1a_) and minor (ivermectin B_1b_) components. It has been determined that both ivermectin B_1a_ and ivermectin B_1b_ components have similar efficacy against veterinary helminths and that these components are too costly to separate at scale. Thus, ivermectin is a racemic mixture of 22,23-dihydroavermectin B_1a_ and 22,23-dihydroavermectin B_1b_ at a ratio of > 80% and < 20%, respectively [14]. Ivermectin B_1a_ (22,23-dihydroavermectin B_1a_) has an ethyl group at C-26, while ivermectin B_1b_ (22,23-dihydroavermectin B_1b_) has a methyl group at C-26 (Fig. 1).

Interestingly, in the snail, *Biomphalaria glabrata*, the ivermectin B_1b_ component was shown to have a snail-lethal effect while ivermectin B_1a_ was inactive [15]. The ivermectin mode of action in mollusks has not been elucidated, but neither is there a complete understanding of ivermectin and GluCl interactions in the *Anopheles* mosquito. The ratios of the ivermectin B_1a_ and ivermectin B_1b_ components can vary between ivermectin manufacturers and batches, and many companies across the globe produce veterinary and human ivermectin. Novel long-lasting formulations of ivermectin are under development for malaria control. Thus, it is important to understand if there are differences in the mosquito-lethal effect of the ivermectin B_1a_ and ivermectin B_1b_ components. Therefore, we have investigated the mosquito-lethal effect of racemic ivermectin, ivermectin B_1a_ and ivermectin B_1b_ against *Anopheles dirus* sensu stricto (*An. dirus* s.s.) and *Anopheles minimus* sensu stricto (*An. minimus* s.s.), the two dominant *Anopheles* malaria vectors in the Greater Mekong Subregion.

## Methods

### Mosquitoes

Mosquitoes for the experiments were reared at the Insecticide Research Unit at the Department of Medical Entomology, Faculty of Tropical Medicine, Mahidol University in Bangkok, Thailand. *Anopheles dirus* (Kaw Mai Khaw strain) mosquitoes were reared as described previously [16], with minor modifications of a rearing environment of 28 ± 2 °C, 80 ± 10% relative humidity and 12/12-h light/dark photoperiod. *Anopheles dirus* larvae were raised at a density of 200 per tray (28 × 35 × 5 cm), with fish food powder (Optimum; Perfect Companion Group Co., Ltd., Bangkok, Thailand) provided daily at quantities of 0.01–0.04 g per tray during the first and second instars and 0.05–0.06 g per tray for the third and fourth instars. *Anopheles minimus* (Saraburi strain) mosquitoes were raised in a similar manner, with the minor modification during larvae rearing, the daily amount of fish food powder provided to 200 larvae per tray was 0.01–0.02 g during the first and second instars, and 0.03–0.04 g per tray for the third and fourth instars. Pupae were transferred to plastic cups and placed inside a cage (30 × 30 × 30 cm). Adult mosquitoes were provided with cotton wool soaked with a 5% sugar solution (Lin, Thai Roong Ruang Sugar Group Co., Ltd., Phetchabun, Thailand) mixed with a 5% multivitamin syrup solution (Seven Seas, PT; Merck Tbk., Jakarta, Indonesia); the cotton wool was changed once a week.

Mosquito larvae used for experiments were reared in the manner described above. Pupae used for experiments were transferred to plastic cylinders (16 diameter × 16.5 cm, diameter × height) filled with 60 ml of water and sealed with a mesh screen. Adult mosquitoes were provided 5% sugar solution mixed with 5% multivitamin syrup solution for the first 48 h post emergence and then with 10% sucrose solution ad libitum until the experiments. Mosquitoes for the experiments were aged 5–7 days post emergence at the time of blood feeding. The mosquitoes were gently transferred via aspiration to 0.5-l cylindrical cardboard containers sealed with mesh screen at the top, with each container holding 40 mosquitoes. The mosquitoes were maintained in an upright incubator maintained at 25 ± 1 °C and 80 ± 10% relative humidity under a 12/12-h light/dark photoperiod. Mosquitoes were sugar-starved but had access to water from 16 to 20 h before their blood meal.

### Compounds

Racemic ivermectin was obtained from Sigma-Aldrich (St. Louis, MO, USA). Powdered ivermectin B_1a_ (95.0% purity) was obtained from Toronto Research Chemicals (Toronto, ON, Canada). Powdered ivermectin B_1b_ (99.27% purity) was obtained from ClearSynth Labs (Mumbai, India). Ivermectin 6 mg tablets (Vermectin^®^) were obtained from Atlantic Laboratories Corp., LTD (Bangkok, Thailand).

### Mosquito blood meal preparation

All compounds were dissolved in dimethylsulfoxide (DMSO) to a concentration of 2 mg/ml and the solutions frozen at − 20 °C until the mosquito feeding experiments. Whole blood was collected from healthy volunteers into sodium heparin tubes on the day of each mosquito membrane feed. Frozen stock solutions of compounds were thawed, and serial dilutions were made in human AB + plasma using glass amber vials. The final plasma stock solution (10 μl) was mixed with blank whole blood (990 μl) to reach the final concentration desired for the mosquito membrane feeding assays. For the experiments with *An. dirus*, ivermectin compounds at concentrations ranging from 2 to 30 nM were fed to the mosquitoes; for *An. minimus*, the concentrations ranged from 0.25 to 4 nM. All blood samples prepared for mosquito feeding contained < 1% organic solvent. Control blood meals were prepared; these consisted of frozen DMSO without ivermectin compounds diluted in plasma to match the highest ratio of DMSO:blood in the compound-containing blood meals.

### Mosquito membrane feeding and mortality assays

At each mosquito membrane feed, whole blood mixed with the different compounds over a range of concentrations were provided to groups of 40 *An. dirus* and 40 *An. minimus* mosquitoes via membrane feeders warmed to 37 °C. Mosquitoes were allowed up to 30 min to membrane feed. After membrane feeding, up to 30 blood-fed mosquitoes per container were gently transferred via aspiration to clean cardboard containers (0.5 l) and the containers maintained in an incubator at 25 ± 1 °C and 80 ± 10% humidity with a 12/12-h light/dark photoperiod. The mosquitoes were provided with 10% sucrose ad libitum. Mosquito survival was monitored daily for 10 days post-blood-feeding, and any dead mosquitoes were removed by aspiration and recorded. Ten days after the blood meal, any remaining mosquitoes were recorded as alive and then frozen.

### Liquid chromatography-mass spectrometry measurements of ivermectin B_1a_ and B_1b_

Racemic ivermectin was dissolved in 50:50 methanol:water to a final concentration of 1 mg/ml, and then diluted 10,000-fold in 40:60 acetonitrile:water. A 5-µl sample of the diluted ivermectin solution (100 ng/ml) was injected into the liquid chromatography-mass spectrometry (LC–MS) system. An ivermectin tablet was crushed in a mortar, and 1 mg of the powder was dissolved in 50:50 methanol:water to a final concentration of 1 mg/ml. The crushed tablet mixture was diluted 10,000-fold in 40:60 acetonitrile:water and sonicated for 30 min. A 5-µl sample of the diluted ivermectin (100 ng/ml) was injected into the LC–MS system. Ivermectin B_1a_ and B_1b_ from the tablet and racemic ivermectin powder were measured to derive the B_1a_ to B_1b_ ratios.

The LC–MS system used was a high-performance liquid chromatography (HPLC) system (Agilent 1260 Quaternary Pump, Agilent 1260 High Performance Autosampler and Agilent 1290 Thermostatic Column Compartment SL; Agilent Technologies, Santa Clara, CA, USA) coupled to a quadrupole time-of-flight mass spectrometer (Q-TOF-MS) (TripleTOF 5600+; Sciex, Framingham, MA, USA), with electrospray ionization (ESI) using a DuoSpray ion source. The HPLC mobile phase was water containing 10 mM ammonium acetate and 0.1% formic acid (mobile phase A) and acetonitrile:water at a 95:5 ratio (*v*/*v*) containing 10 mM ammonium acetate and 0.1% formic acid (mobile phase B). LC vials were kept in the autosampler at 6 °C during analysis. Sample mixtures were injected onto a C18 reversed-phase column (Acquity UPLC HSS T3, 2.1 × 100 mm, 1.8 μm; Waters Corp., Milford, MA, USA) protected by a precolumn (Acquity UPLC HSS T3, 2.1 × 5 mm, 1.8 μm; Waters Corp.) for separation at a flow rate of 0.3 ml/min at 40 °C. The HPLC elution gradient started at 40% mobile phase B for 2.0 min (0–2.0 min), followed by 40–80% B for 2.0 min (2.0–4.0 min), 80–100% B for 5.0 min (4.0–9.0 min), 100% B for 5.0 min (9.0–14.0 min), 100–40% B for 0.1 min (14.0–14.1), and 40% B for 3.9 min (14.1–18.0 min). The HPLC-Q-TOF-MS system, mass ion chromatogram and mass spectra were acquired by Analyst™ Software version 1.7 (Sciex). The Q-TOF-MS was operated in the ESI-positive mode, ion source gases 1 and 2 at 40 psi each, curtain gas at 30 psi, ion spray voltage at 4500 V, source temperature at 350 °C and declustering potential at 120 V. Data were acquired in the informative-dependent acquisition mode, composed of a TOF-MS scan and 10 dependent product ion scans in the high sensitivity mode with dynamic background subtraction. The mass range of TOF-MS scan was *m*/*z* 100–1000 and product ion scan was *m*/*z* 50–1000. Quantification of the level of ivermectin B_1a_ and B_1b_ were performed by MultiQuant™ Software (Sciex) with a high-resolution TOF-MS scan mode.

### Statistical analyses

The lethal concentrations that kill 50% and 90% of mosquitoes (LC_50_ and LC_90_, respectively) were estimated using a normalized four-variable concentration–response analysis (IC_50_ [half-maximal inhibitory concentration], Hill [Hill-Slope], E_MIN_ [point of minimum mortality], and E_MAX_ [point of maximum mortality]). All parameters were estimated from the observed data, except E_MAX_, which was assumed to reach 100% at infinite concentrations. The 95% confidence intervals (95% CI) around point estimates were derived using a symmetrical (asymptotic) approximation. All mosquito survival analyses were performed with GraphPad Prism v.10.2 (GraphPad Software Inc., San Diego, CA, USA).

## Results

### Impact of ivermectin compounds on mosquito mortality

**Fig. 2:**
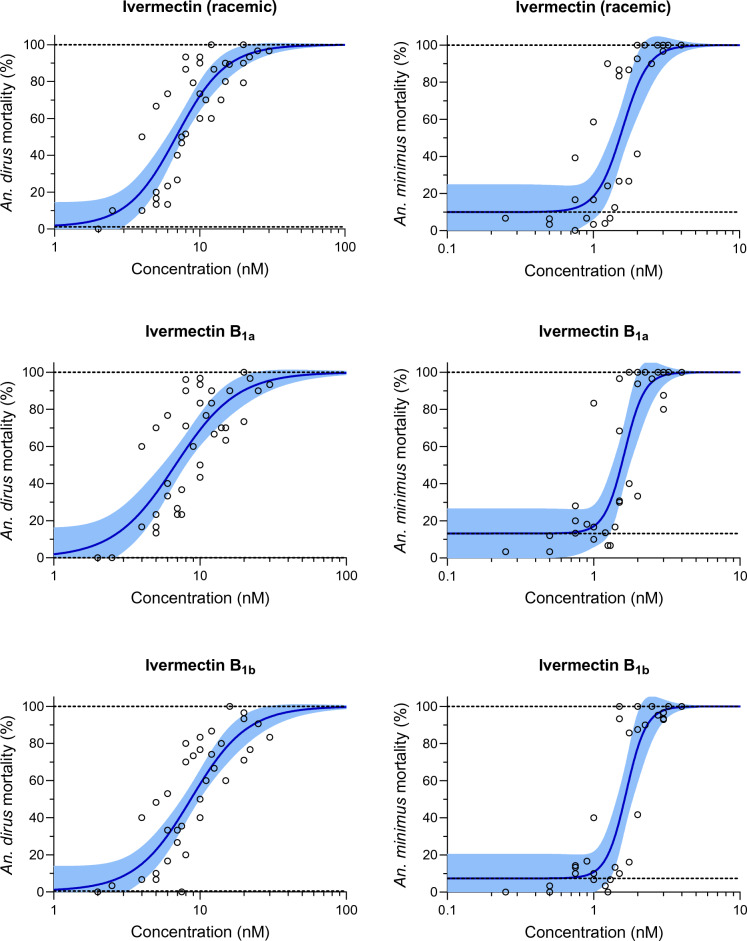
Mosquito mortality results for racemic ivermectin, ivermectin B_1a_ and ivermectin B_1b_. Mortality results for *Anopheles dirus* (left panels) and *Anopheles minimus* (right panels) when mosquitoes fed on blood containing racemic ivermectin (upper panels), ivermectin B_1a_ (middle panels) or ivermectin B_1b_ (lower panels). Circles represent cumulative mosquito mortality at 10 days after blood meal ingestion. Solid blue lines represent the mean concentration–response relationship, and the blue-shaded area represents the 95% confidence interval associated with the nonlinear fit. Dashed black lines represent the fixed maximum effects of 100% mortality (top) and the estimated minimum effect (bottom) associated with baseline mortality observed from control mosquitoes

Five replicates with a total of 3869 *An. dirus* mosquitoes were used to calculate the LC_50_ and LC_90_ values for ivermectin compounds, including racemic ivermectin (*n* = 1286), ivermectin B_1a_ (*n* = 1286) and ivermectin B_1b_ (*n* = 1297). Four replicates with a total of 2931 *An. minimus* were used to calculate the LC_50_ and LC_90_ values for ivermectin compounds, including racemic ivermectin (*n* = 1042), ivermectin B_1a_ (*n* = 933) and ivermectin B_1b_ (*n* = 956). All three ivermectin compounds were found to have similar mosquito-lethal effects for both *An. dirus* and *An. minimus* (Fig. 2; Table 1). The susceptibility of *An. minimus* was four- to five-fold lower than that of *An. dirus* (Table 1).

**Table 1:** *Anopheles dirus* and *Anopheles minimus* LC_50_ and LC_90_ values for ivermectin compounds

*Anopheles* species	Compound	LC_50_ [95% CI] (nM)	LC_90_ [95% CI] (nM)
*An. dirus*	Racemic ivermectin	6.93 [5.72–8.15]	16.41 [11.68–21.14]
	Ivermectin B_1a_	6.79 [5.11–8.47]	19.77 [11.66–27.88]
	Ivermectin B_1b_	8.70 [7.03–10.37]	22.97 [14.89–31.04]
*An. minimus*	Racemic ivermectin	1.59 [1.34–1.83]	2.49 [1.70–3.27]
	Ivermectin B_1a_	1.63 [1.41–1.84]	2.30 [1.66–2.94]
	Ivermectin B_1b_	1.67 [1.47–1.87]	2.36 [1.75–2.97]

*CI* Confidence interval, *LC50, LC90* lethal concentration that kills 50% and 90% of mosquitoes

Table 1 presents the LC_50_ and LC_90_ of either *An. dirus* or *An. minimus* at 10 days after a blood meal containing racemic ivermectin, ivermectin B_1a_ or ivermectin B_1b_.

### Ivermectin B_1a_ and ivermectin B_1b_ content

Fifteen injection replications were performed to determine the average peak ratios from commercially available racemic ivermectin powder. The analysis showed that the powder consisted of 98.84% ivermectin B_1a_ and 1.16% ivermectin B_1b_, with a standard deviation of 0.1958%. Fifteen injection replications were performed to determine the average peak ratios from marketed ivermectin tablets. The analysis showed that the tablets contained 98.55% ivermectin B_1a_ and 1.45% ivermectin B_1b_, with a standard deviation of 0.3977%.

## Discussion

These in vitro results demonstrate that ivermectin B_1a_ and ivermectin B_1b_ had equivalent mosquito-lethal effects in both *An. dirus* and *An. minimus* (Fig. 2; Table 1). That both components showed similar mosquito-lethal effects is extremely favorable as it would be cost prohibitive to separate ivermectin B_1a_ and ivermectin B_1b_ at scale. For malaria control, this finding also suggests that there should be no concerns regarding the ratios of ivermectin B_1a_ and ivermectin B_1b_ components between ivermectin batches and manufacturers. This similarity is important when developing novel formulations of ivermectin for malaria control. As ivermectin metabolites have mosquito-lethal effect [2, 3], these metabolites could arise from either ivermectin B_1a_ or ivermectin B_1b_ components [17–19].

In *C. elegans*, ivermectin binds to GluCl channel transmembrane helixes (M1–M4) as follows: hydrogen bond at C1 to M1, hydrogen bond at O10 to M2, Van der Waals forces at C48 to M2 and hydrogen bond at O14 to M3. The mode of action is purported to occur when ivermectin binds via Van der Waals forces at C32, C33, C35 to the GluCl extracellular M2-M3 loop (Fig. 1). Ivermectin attachment to the M2-M3 loop shifts position of the M2 helix and opens the GluCl channel [7]. As the C-26 ethyl (ivermectin B_1a_) and C-26 methyl (ivermectin B_1b_) groups are not located near the GluCl M2-M3 loop binding points, nor close to other GluCl channel binding points (Fig. 1), this suggests that there would be no mosquito-lethal difference between ivermectin B_1a_ and ivermectin B_1b_, which is validated by the findings of the present study (Fig. 2). Snails are protostome invertebrates and thus have GluCl channels. Investigations, while limited in number, have demonstrated the snail-lethal effect of ivermectin on *B. glabrata* [15, 20]. However, there has been no investigation of the ivermectin-GluCl interaction in the snail to understand why ivermectin B_1b_ would have snail-lethal effect while ivermectin B_1a_ has none.

Regarding the purity of the compounds administered, ivermectin B_1a_ was 95% pure and ivermectin B_1b_ was 99.27% pure, but we cannot be certain what the other 5% and 1% of the contents of the compounds provided. However, if ivermectin B_1b_ was the only component with a mosquito-lethal effect, as seen in snails, then we would expect greatly reduced mosquito-killing effect from the ivermectin B_1a_ experiments compared to ivermectin B_1b_. Ivermectin is reported to be a racemic mixture of > 80% ivermectin B_1a_ and < 20% ivermectin B_1b_. However, the racemic ivermectin powder used in most of the *Anopheles* ivermectin susceptibility evaluations was analyzed in the present study and found to contain 98.84% ivermectin B_1a_ and 1.16% ivermectin B_1b_. Furthermore, the human oral ivermectin tablets evaluated here contained 98.55% ivermectin B_1a_ and 1.45% ivermectin B_1b_. These points further stress that it is ideal that ivermectin B_1a_ and ivermectin B_1b_ have comparable *Anopheles* mosquito-lethal effects as there is no need to modify the manufacturing process for ivermectin used for malaria control.

## Conclusions

The results presented here demonstrated an equal *Anopheles* mosquito-lethal effect of ivermectin B_1a_ and ivermectin B_1b_ components. Thus, there is no need to modify the manufacturing process for ivermectin used for malaria control. Variations in ivermectin B_1a_ and ivermectin B_1b_ ratios between ivermectin batches, manufacturers and future formulations should not impact the mosquito-lethal efficacy of ivermectin.

## Data Availability

The data supporting the findings of the study must be available within the article and/or its supplementary materials, or deposited in a publicly available database.

## Abbreviations

DMSO: Dimethylsulfoxide
ESI: Electrospray ionization
GluCl: Glutamate-gated chloride
HPLC: High performance liquid chromatography
LC–MS: Liquid chromatography-mass spectrometry
LC_50_: Lethal concentration that kills 50% of mosquitoes
MDA: Mass drug administration
Q-TOF-MS: Quadrupole time-of-flight mass spectrometry

## References

[1] Billingsley P, Binka F, Chaccour C, Foy B, Gold S, Gonzalez-Silva M (2020). A roadmap for the development of ivermectin as a complementary malaria vector control tool. Am J Trop Med Hyg.

[2] Kobylinski K, Tipthara P, Wamaket N, Chainarin S, Kullasakboonsri R, Sriwichai P (2023). Ivermectin metabolites reduce *Anopheles* survival. Sci Rep.

[3] Kern C, Müller P, Chaccour C, Liechti M, Hammann F, Duthaler U (2023). Pharmacokinetics of ivermectin metabolites and their activity against *Anopheles stephensi* mosquitoes. Malar J.

[4] Cully D, Vassilatis D, Liu K, Paress P, Van der Ploeg L, Schaeffer J (1994). Cloning of an avermectin-sensitive glutamate-gated chloride channel from *Caenorhabditis elegans*. Nature.

[5] Cully D, Paress P, Liu K, Schaeffer J, Arena J (1996). Identification of a *Drosophila melanogaster* glutamate-gated chloride channel sensitive to the antiparasitic agent avermectin. J Biol Chem.

[6] Kane N, Hirschberg B, Qian S, Hunt D, Thomas B, Brochu R (2000). Drug-resistant *Drosophila* indicate glutamate-gated chloride channels are targets for the antiparasitics nodulisporic acid and ivermectin. Proc Natl Acad Sci USA.

[7] Hibbs R, Gouaux E (2011). Principles of activation and permeation in an anion-selective Cys-loop receptor. Nature.

[8] Albers-Schonberg G, Arison B, Chabala J, Douglas A, Eskola P, Fisher M (1981). Avermectins—structure determination. J Am Chem Soc.

[9] Yoon Y, Kim E, Hwang Y, Choi C (2004). Avermectin: biochemical and molecular basis of its biosynthesis and regulation. Appl Microbiol Biotechnol.

[10] Wei L, Wei G, Zhang H, Wang P, Du Y (2005). Synthesis of new, potent avermectin-like insecticidal agents. Carbohydr Res.

[11] Pitterna T, Cassayre J, Hüter O, Jung P, Maienfisch P, Kessabi F (2009). New ventures in the chemistry of avermectins. Bioorg Med Chem.

[12] Chabala J, Mrozik H, Tolman R, Eskola P, Lusi A, Peterson L (1980). Ivermectin, a new broad-spectrum antiparasitic agent. J Med Chem.

[13] Campbell W (2012). History of avermectin and ivermectin, with notes on the history of other macrocyclic lactone antiparasitic agents. Curr Pharm Biotechnol.

[14] Campbell W, Fisher M, Stapley E, Albers-Schönberg G, Jacob T (1983). Ivermectin: a potent new antiparasitic agent. Science.

[15] Katz N, Araújo N, Coelho P, Morel C, Linde-Arias A, Yamada T (2017). Ivermectin efficacy against *Biomphalaria*, intermediate host snail vectors of Schistosomiasis. J Antibiot.

[16] Sutthanont N, Sudsawang M, Phanpoowong T, Sriwichai P, Ruangsittichai J, Rotejanaprasert C (2022). Effectiveness of herbal essential oils as single and combined repellents against *Aedes aegypti*, *Anopheles dirus* and *Culex quinquefasciatus* (Diptera: Culicidae). Insects.

[17] Chiu S, Sestokas E, Taub R, Buhs R, Green M, Sestokas R (1986). Metabolic disposition of ivermectin in tissues of cattle, sheep, and rats. Drug Metab Dispos.

[18] Chiu S, Taub R, Sestokas E, Lu A, Jacob T (1987). Comparative in vivo and in vitro metabolism of ivermectin in steers, sheep, swine, and rat. Drug Metab Rev.

[19] Chiu S, Carlin J, Taub R, Sestokas E, Zweig J, Vandenheuvel W (1988). Comparative metabolic disposition of ivermectin in fat tissues of cattle, sheep, and rats. Drug Metab Dispos.

[20] Matha V, Weiser J (1988). Molluscicidal effect of ivermectin on *Biomphalaria glabrata*. J Invertebr Pathol.

